# Residual Salivary Secretion Ability May Be a Useful Marker for Differential Diagnosis in Autoimmune Diseases

**DOI:** 10.1155/2014/534261

**Published:** 2014-11-20

**Authors:** Etsuko Maeshima, Hiroya Koshiba, Kanako Furukawa, Shinichiro Maeshima, Wataru Sakamoto

**Affiliations:** ^1^Department of Health and Sport Management, Osaka University of Health and Sport Sciences, 1-1 Asashirodai, Kumatori-cho, Sennan-gun, Osaka 590-0496, Japan; ^2^Third Department of Internal Medicine, Wakayama Medical University, 811-1 Kimiidera, Wakayama City, Wakayama 641-8509, Japan; ^3^Department of Internal Medicine, Kainan Municipal Medical Center, Kainan City, Wakayama 642-0002, Japan; ^4^Department of Rehabilitation, Nanakuri Sanatorium, Fujita Health University, Tsu City, Mie 514-1295, Japan; ^5^Serotec Laboratory, Ebetsu City, Hokkaido 069-0822, Japan

## Abstract

*Background.* We have elucidated decreased resting salivary flow in approximately 60% of patients with autoimmune diseases not complicated by Sjögren syndrome (SjS). In this study, salivary stimulation tests using capsaicin were performed to examine residual salivary secretion ability in patients with autoimmune diseases. *Materials and Methods.* Fifty-eight patients were divided into three groups: patients with primary or secondary SjS (SjS group), patients with systemic sclerosis not complicated by SjS (SSc group), and patients with other autoimmune diseases (non-SjS/non-SSc group). Simple filter paper and filter paper containing capsaicin were used to evaluate salivary flow rates. *Results.* Resting salivary flow rates were significantly lower in the SjS and SSc groups than in the non-SjS/non-SSc group but did not differ significantly between the SjS and SSc groups. Capsaicin-stimulated salivary flow rates were significantly lower in the SjS and SSc groups than in the non-SjS/non-SSc group, but not significantly different between the SjS and SSc groups. In the non-SjS/non-SSc group, salivary flow rates increased after capsaicin stimulation to the threshold level for determination of salivary gland dysfunction, whereas no improvement was observed in the SjS and SSc groups. *Conclusion.* Residual salivary secretion ability may be a useful marker for differential diagnosis in autoimmune diseases.

## 1. Introduction

Xerostomia is a disease caused by decreased salivary flow due to various factors [1]. It causes inflammation of the oral cavity, resulting in halitosis, stomatitis, dental caries, and periodontitis. Thus saliva plays an important role to maintain the oral hygiene. Recent studies have shown that the oral hygienic environment may influence the incidence of cardiac diseases, respiratory infection, and autoimmune diseases [2]. Regarding autoimmune diseases in particular, periodontitis is associated with the incidence and disease progression of rheumatoid arthritis (RA) [3–7]. Systemic sclerosis (SSc) has been known to be complicated by xerostomia. Possible reasons for this are concomitant Sjögren syndrome (SjS) and salivary gland fibrosis due to SSc itself [8, 9]. Indeed, we observed decreased salivary flow in approximately 60% of patients with autoimmune diseases not complicated by SjS; among those patients, impaired salivary flow was most often observed in patients with SSc [10]. Moreover, morbidity of dental caries and periodontitis is high in patients with SSc [11, 12]. Severe structural destruction of the salivary glands is often observed in SjS. In such cases, the salivary glands do not express adequate salivary responses even when stimulated. However, if decreased salivary flow is attributable to slight structural destruction or functional disorder of the salivary glands, it is inferred that residual salivary gland function is preserved and salivary responses to stimulation will be induced.

Capsaicin, a component of hot pepper that is widely consumed as a spice, stimulates salivation through the gustatory reflex, the trigeminal-parasympathetic reflex [13, 14]. Therefore, capsaicin should also stimulate salivation and increase salivary flow in patients with autoimmune diseases. However, salivary responses to capsaicin in patients with autoimmune diseases have not been extensively studied [15].

In the present study, we hypothesized that capsaicin-stimulated salivary flow rates do not increase in SjS and SSc. Therefore, salivary stimulation tests were performed in order to examine the salivary responses to capsaicin and the residual salivary secretion ability in patients with autoimmune diseases.

## 2. Materials and Methods

### 2.1. Study Design and Subjects

Out of 160 patients who understood the main purpose of this study and provided written informed consent, 58 patients (3 men and 55 women) with decreased resting salivary flow were included. All participants in this study signed an informed consent. This study was approved by the Ethics Committee of Osaka University of Health and Sport Sciences based on the Declaration of Helsinki.

Because salivation in patients with autoimmune diseases is affected by SjS [16] and SSc [17], the subjects were divided into three groups: patients with primary or secondary SjS (SjS group, *n* = 26), patients with SSc not complicated by SjS (SSc group, *n* = 12), and patients with autoimmune diseases not complicated by SjS and SSc (non-SjS/non-SSc group, *n* = 20). The ages in each group, expressed as mean ± standard deviation (SD), were 55.1 ± 13.3 years, 64.5 ± 7.9 years, and 49.4 ± 14.1 years, respectively. The breakdown of diseases was as follows: the SjS group consisted of six patients with primary SjS and 20 with secondary SjS (three with RA [18], 10 with systemic lupus erythematosus (SLE) [19], one with polymyositis (PM) [20], four with mixed connective tissue disease (MCTD) [21], one with adult-onset Still's disease (AOSD) [22], and one with fibromyalgia syndrome [23]); the non-SjS/non-SSc group consisted of four patients with RA, nine with SLE, two with PM, one with MCTD, three with AOSD, and one with Behcet's disease [24].

### 2.2. Evaluation of Resting and Stimulated Salivary Flow Rates

Filter paper was used for measurement of resting salivary flow rates. Three or more colored spots on the filter paper correspond to a salivary flow rate of 200 *μ*L/min or less [25], defined as decreased salivary flow, and the presence of impaired salivary flow is determined [26]. For salivary stimulation test using capsaicin, we used a method described by Kanehira et al. using filter paper containing capsaicin [15]. The filter paper is a strip, 21 mm in width and 67 mm in length, which is developed by combining the principle of paper chromatography with the iodine-starch reaction; capsaicin is applied to the tip of the strip. A portion of the strip approximately 47 mm from this tip is inserted under the tongue, and the mouth is kept closed for 2 minutes. The filter paper is removed from under the tongue 2 minutes after insertion and reacted with a color-developing reagent, and then the number of colored spots is counted. The coloring agent was prepared from a solution of 31% hydrogen peroxide (Kanto Chemical Co., Inc.), ethyl alcohol (Kanto Chemical Co., Inc.), and distilled water at ratio of 1 : 7 : 1 [25]. When salivary flow is low, the number of colored spots increases. We explained to the subjects beforehand that they should brush their teeth 2 hours before the test, refrain from eating anything after brushing, and refrain from drinking anything for 1 hour before the test. Although the filter paper is odorless, a very mild irritation (tingling sensation) may be felt when the filter paper containing capsaicin is inserted under the tongue.

### 2.3. Statistical Analysis

The number of colored spots in each group was expressed as mean ± SD. For comparison of the numbers of colored spots among the groups, analysis of variance was performed. When statistically significant differences were observed, post hoc tests were performed to determine significant differences between the groups. For comparison between resting and capsaicin-stimulated salivary flow rates within each group, Wilcoxon signed rank tests were performed. Statistical significance was defined as *P* < 0.05.

## 3. Results

### 3.1. Intergroup Comparison of Colored Spots' Number on the Filter Paper

**Figure 1:**
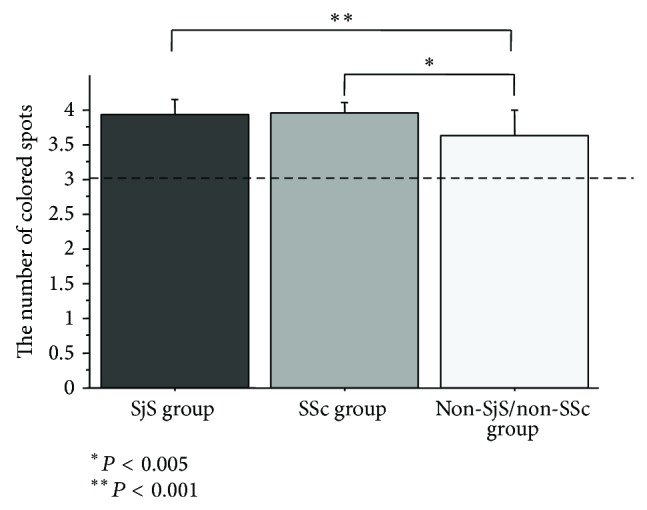
Intergroup comparison of resting salivary flow. The number of colored spots was significantly different among the three groups (*P* < 0.001). Although the numbers of colored spots in the SjS and the SSc groups were both significantly larger than that in the non-SjS/non-SSc group (*P* < 0.001 and *P* < 0.005, resp.), no significant difference was observed between the SjS and the SSc groups.

The number of colored spots was 3.9 ± 0.2 in the SjS group, 4.0 ± 0.1 in the SSc group, and 3.6 ± 0.4 in the non-SjS/non-SSc group, with significant differences among the three groups (*P* < 0.001). Although the numbers of colored spots in the SjS and the SSc groups were both significantly larger than that in the non-SjS/non-SSc group (*P* < 0.001 and *P* < 0.005, resp.), no significant difference was observed between the SjS and the SSc groups (Figure 1).

### 3.2. Intergroup Comparison of Colored Spots' Number on the Filter Paper Containing Capsaicin

**Figure 2:**
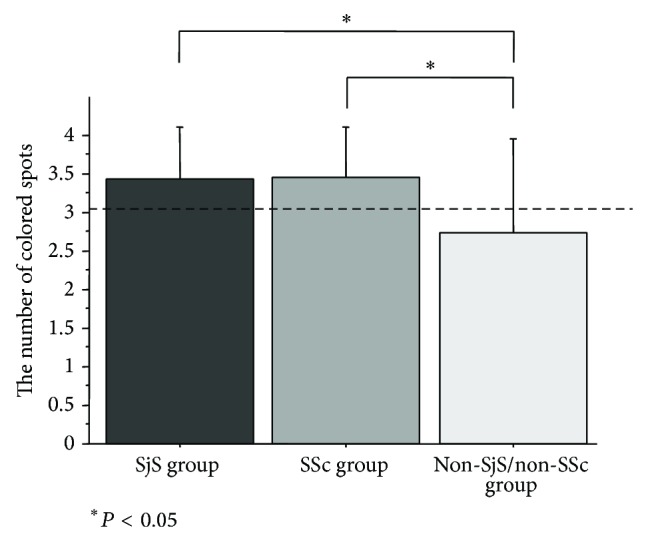
Intergroup comparison of capsaicin-stimulated salivary flow. The number of colored spots was significant differences among the three groups (*P* < 0.005). Although the numbers of colored spots in the SjS and the SSc groups were both significantly larger than that in the non-SjS/non-SSc group (*P* < 0.005 and *P* < 0.005, resp.), no significant difference was observed between the SjS and the SSc groups.

The number of colored spots was 3.4 ± 0.7 in the SjS group, 3.5 ± 0.7 in the SSc group, and 2.7 ± 1.2 in the non-SjS/non-SSc group, with significant differences among the three groups (*P* < 0.005). Although the numbers of colored spots in the SjS and the SSc groups were both significantly larger than that in the non-SjS/non-SSc group (*P* < 0.005 and *P* < 0.005, resp.), no significant difference was observed between the SjS and the SSc groups (Figure 2).

### 3.3. Comparison between Resting and Capsaicin-Stimulated Colored Spots' Number within Each Group

The number of colored spots significantly decreased after stimulation with capsaicin in all groups (SjS, *P* < 0.001; SSc, *P* < 0.05; non-SjS/non-SSc, *P* < 0.01). Thus, stimulation with capsaicin increased salivary flow. However, the numbers of colored spots in the SjS and the SSc groups did not decrease below the threshold for determination of impaired salivary flow, whereas the number in the non-SjS/non-SSc group decreased to that level (Table 1).

## 4. Discussion

Saliva plays an important role in maintaining oral health. Decreased salivary flow causes various oral diseases, such as infection of the oral cavity and periodontitis [27]. In autoimmune diseases, periodontitis may be associated with the incidence and disease progression of RA [3–7]. Furthermore, recent studies have shown that growth factors considered as predisposing factors for periodontitis, such as TGF-*β* and VEGF, are also important in the etiology of SSc and that periodontitis and SSc share etiological aspects [28]. Because biomarkers in saliva are useful for diagnosing periodontal and oral diseases, breast cancer, SjS, and so forth [29, 30], not only is saliva important for the maintenance of oral hygiene, but it has also attracted attention as a possible indicator for diagnosis of systemic diseases.

We previously reported that a decrease in resting salivary flow is also observed in patients with autoimmune diseases not complicated by SjS [10]. In this study, we performed salivary stimulation tests using capsaicin in patients with autoimmune diseases who exhibited decreased resting salivary flow rates. Our results showed that salivary responses to the stimulation were inadequate in the SjS and the SSc groups but adequate in the non-SjS/non-SSc group. Capsaicin, which is orally consumed as a spice, stimulates salivation through the trigeminal-parasympathetic reflex [13, 14]. However, the results presented here reveal that capsaicin does not stimulate salivation in patients with the autoimmune diseases SjS and SSc. Based on these results, patients with SjS or SSc have severely impaired salivary flow and little residual salivary gland function. On the other hand, patients with other autoimmune diseases may have slight or mild impairment of salivary flow and maintain residual salivary gland function.

Salivary secretion follows cholinergic stimulation of muscarinic type 3 receptors (M3R) [31, 32]. Anti-M3 muscarinic acetylcholine receptor antibodies (anti-M3R antibodies) have been shown in patients with SjS [33–35]. It has not been concluded how much the antibodies are involved in salivary gland dysfunction in SjS. However, being the cause that salivary secretion did not increase after capsaicin stimulation in SjS in the present study, anti-M3R antibodies may also play a role as well as the lymphocytic infiltration and destruction of salivary glands [36]. Fibrosis of the salivary glands has been reported in SSc [8]. Thus, we postulated that patients with these two diseases might have failed to respond to salivary stimulation test because they had structural disorders of the salivary glands. On the other hand, because salivary responses to capsaicin stimulation were observed in patients with other autoimmune diseases, we speculate that the disorders of the salivary glands in those patients are functional rather than structural. Although it is well established that salivary flow is reduced in SjS and SSc, there have been very few reports of studies on salivary responses to stimulation [15]. The results of this study, which revealed decreased salivary responses to stimulation in SjS and SSc, will be important in consideration of future methods for improving the oral hygienic environment in both diseases.

Many patients with SjS notice inconvenience in eating or talking due to oral dryness caused by decreased salivary flow or visit dental clinics because they suffer from pain of the tongue or dental caries. However, the proportion of people who regularly visit a dental clinic is significantly smaller in patients with SSc than in people without SSc [12]. It is also possible that patients with SSc cannot receive adequate dental care due to trismus. The salivary stimulation test performed in this study, which uses filter paper, is simple and enables evaluation of salivary responses to stimulation without causing pain to patients at low cost. We believe that it is important to detect salivary gland dysfunction using this method in the early phase of disease and to try to maintain oral health in terms of prevention of disease development and inhibition of disease progression.

## 5. Conclusion

We performed salivary stimulation tests using filter paper containing capsaicin in patients with autoimmune diseases. Capsaicin did not stimulate salivation in patients with SjS and SSc. The results revealed that patients with SjS and SSc have severely impaired salivary flow and residual salivary gland function. We suggest that residual salivary secretion ability may be a useful marker for differential diagnosis in autoimmune diseases.

## Figures and Tables

**Table 1:** Differences between resting and capsaicin-stimulated colored spots' number in 3 groups.

	Number of colored spots on the filter paper	Number of colored spots on the filter paper containing capsaicin	
SjS group (*n* = 26)	3.9 ± 0.2	3.4 ± 0.7	*P* < 0.001
SSc group (*n* = 12)	4.0 ± 0.1	3.5 ± 0.7	*P* < 0.05
Non-SjS/non-SSc group (*n* = 20)	3.6 ± 0.4	2.7 ± 1.2	*P* < 0.01

Mean ± SD.

## References

[1] Keitel W., Spieler C. (1989). The Saxon test for objective assessment of xerostomia. A contribution to the diagnosis of Sjögren's syndrome. *Zeitschrift für die Gesamte Innere Medizin und Ihre Grenzgebiete*.

[2] Manjunath B. C., Praveen K., Chandrashekar B. R., Rani R. M., Bhalla A. (2011). Periodontal infections: a risk factor for various systemic diseases. *National Medical Journal of India*.

[3] Detert J., Pischon N., Burmester G. R., Buttgereit F. (2010). The association between rheumatoid arthritis and periodontal disease. *Arthritis Research and Therapy*.

[4] Berthelot J.-M., Le Goff B. (2010). Rheumatoid arthritis and periodontal disease. *Joint Bone Spine*.

[5] Hoovestol R. A., Mikuls T. R. (2011). Environmental exposures and rheumatoid arthritis risk. *Current Rheumatology Reports*.

[6] Routsias J. G., Goules J. D., Goules A., Charalampakis G., Pikazis D. (2011). Autopathogenic correlation of periodontitis and rheumatoid arthritis. *Rheumatology*.

[7] Persson G. R. (2012). Rheumatoid arthritis and periodontitis-inflammatory and infectious connections. Review of the literature. *Journal of Oral Microbiology*.

[8] Cipoletti J. F., Buckingham R. B., Barnes E. L. (1977). Sjogren's syndrome in progressive systemic sclerosis. *Annals of Internal Medicine*.

[9] Drosos A. A., Andonopoulos A. P., Costopoulos J. S., Stavropoulos E. D., Papadimitriou C. S., Moutsopoulos M. (1988). Sjogren's syndrome in progressive systemic sclerosis. *Journal of Rheumatology*.

[10] Maeshima E., Furukawa K., Maeshima S., Koshiba H., Sakamoto W. (2013). Hyposalivation in autoimmune diseases. *Rheumatology International*.

[11] Wood R. E., Lee P. (1988). Analysis of the oral manifestations of systemic sclerosis (scleroderma). *Oral Surgery Oral Medicine and Oral Pathology*.

[12] Chu C. H., Yeung C. M. K., Lai I. A., Leung W. K., Mok M. Y. (2011). Oral health of Chinese people with systemic sclerosis. *Clinical Oral Investigations*.

[13] Duner-Engstrom M., Fredholm B. B., Larsson O. (1986). Autonomic mechanisms underlying capsaicin induced oral sensations and salivation in man. *Journal of Physiology*.

[14] Nasrawi C. W., Pangborn R. M. (1990). Temporal gustatory and salivary responses to capsaicin upon repeated stimulation. *Physiology and Behavior*.

[15] Kanehira T., Yamaguchi T., Asano K. (2011). A screening test for capsaicin-stimulated salivary flow using filter paper: a study for diagnosis of hyposalivation with a complaint of dry mouth. *Oral Surgery, Oral Medicine, Oral Pathology, Oral Radiology and Endodontology*.

[16] Shiboski S. C., Shiboski C. H., Criswell L. A., Baer A. N., Challacombe S., Lanfranchi H., Schiødt M., Umehara H., Vivino F., Zhao Y., Dong Y., Greenspan D., Heidenreich A. M., Helin P., Kirkham B., Kitagawa K., Larkin G., Li M., Lietman T., Lindegaard J., McNamara N., Sack K., Shirlaw P., Sugai S., Vollenweider C., Whitcher J., Wu A., Zhang S., Zhang W., Greenspan J. S., Daniels T. E. (2012). American College of Rheumatology classification criteria for Sjögren’s syndrome: a data-driven, expert consensus approach in the Sjogren’s international collaborative clinical alliance cohort. *Arthritis Care and Research*.

[17] Masi A. T. (1980). Preliminary criteria for the classification of systemic sclerosis (scleroderma). *Arthritis & Rheumatism*.

[18] Aletaha D., Neogi T., Silman A. J., Funovits J., Felson D. T., Bingham C. O., Birnbaum N. S., Burmester G. R., Bykerk V. P., Cohen M. D., Combe B., Costenbader K. H., Dougados M., Emery P., Ferraccioli G., Hazes J. M. W., Hobbs K., Huizinga T. W. J., Kavanaugh A., Kay J., Kvien T. K., Laing T., Mease P., Ménard H. A., Moreland L. W., Naden R. L., Pincus T., Smolen J. S., Stanislawska-Biernat E., Symmons D., Tak P. P., Upchurch K. S., Vencovský J., Wolfe F., Hawker G. (2010). 2010 Rheumatoid arthritis classification criteria: an American College of Rheumatology/European League Against Rheumatism collaborative initiative. *Arthritis and Rheumatism*.

[19] Tan E. M., Cohen A. S., Fries J. F. (1982). The 1982 revised criteria for the classification of systemic lupus erythrematosus. *Arthritis & Rheumatism*.

[20] Bohan A., Peter J. B. (1975). Polymyositis and dermatomyositis. *The New England Journal of Medicine*.

[21] Kasukawa R. (1999). Mixed connective tissue disease. *Internal Medicine*.

[22] Yamaguchi M., Ohta A., Tsunematsu T., Kasukawa R., Mizushima Y., Kashiwagi H., Kashiwazaki S., Tanimoto K., Matsumoto Y., Ota T., Akizuki M. (1992). Preliminary criteria for classification of adult Still's disease. *Journal of Rheumatology*.

[23] Wolfe F., Smythe H. A., Yunus M. B., Bennett R. M., Bombardier C., Goldenberg D. L., Tugwell P., Campbell S. M., Abeles M., Clark P., Fam A. G., Farber S. J., Fiechtner J. J., Franklin C. M., Gatter R. A., Hamaty D., Lessard J., Lichtbroun A. S., Masi A. T. (1990). The american college of rheumatology 1990 criteria for the classification of fibromyalgia. *Arthritis and Rheumatism*.

[24] International Study Group for Behçet’s Disease (1990). Criteria for diagnosis of Behçet’s disease. *The Lancet*.

[25] Kanehira T., Yamaguchi T., Takehara J., Kashiwazaki H., Abe T., Morita M., Asano K., Fujii Y., Sakamoto W. (2009). A pilot study of a simple screening technique for estimation of salivary flow. *Oral Surgery, Oral Medicine, Oral Pathology, Oral Radiology and Endodontology*.

[26] Longman L. P., McCracken C. F. M., Higham S. M., Field E. A. (2000). The clinical assessment of oral dryness is a significant predictor of salivary gland hypofunction. *Oral Diseases*.

[27] Hopcraft M. S., Tan C. (2010). Xerostomia: an update for clinicians. *Australian dental journal*.

[28] Matarese G., Isola G., Anastasi G. P. (2012). Immunohistochemical analysis of TGF-*β*1 and VEGF in gingival and periodontal tissues: a role of these biomarkers in the pathogenesis of scleroderma and periodontal disease. *International Journal of Molecular Medicine*.

[29] Patil P. B., Patil B. R. (2011). Saliva: a diagnostic biomarker of periodontal diseases. *Journal of Indian Society of Periodontology*.

[30] Liu J., Duan Y. (2012). Saliva: a potential media for disease diagnostics and monitoring. *Oral Oncology*.

[31] Nakamura T., Matsui M., Uchida K. (2004). M_3_ muscarinic acetylcholine receptor plays a critical role in parasympathetic control of salivation in mice. *The Journal of Physiology*.

[32] Matsui M., Motomura D., Karasawa H., Fujikawa T., Jiang J., Komiya Y., Takahashi S.-I., Taketo M. M. (2000). Multiple functional defects in peripheral autonomic organs in mice lacking muscarinic acetylcholine receptor gene for the M3 subtype. *Proceedings of the National Academy of Sciences of the United States of America*.

[33] Dawson L., Tobin A., Smith P., Gordon T. (2005). Antimuscarinic antibodies in Sjögren's syndrome: where are we, and where are we going?. *Arthritis and Rheumatism*.

[34] Dawson L. J., Stanbury J., Venn N., Hasdimir B., Rogers S. N., Smith P. M. (2006). Antimuscarinic antibodies in primary Sjögren's syndrome reversibly inhibit the mechanism of fluid secretion by human submandibular salivary acinar cells. *Arthritis & Rheumatism*.

[35] Sumida T., Tsuboi H., Iizuka M., Asashima H., Matsumoto I. (2013). Anti-M3 muscarinic acetylcholine receptor antibodies in patients with Sjögren's syndrome. *Modern Rheumatology*.

[36] Daniels T. E., Aufdermonte T. B., Greenspan J. S., Talal N., Moutsopolous H. M., Kassan S. (1987). Histopathology of Sjögren’s syndrome. *Sjögren’s Syndrome: Clinical and Immunological Aspects*.

